# Analysis of Prescription Pattern of Anti-diabetic Medications in a Teaching Hospital in North India

**DOI:** 10.7759/cureus.63343

**Published:** 2024-06-28

**Authors:** Hemali Jha, Tauseef Ahmad Khan, Nida Khan, Ghizal Fatima

**Affiliations:** 1 Internal Medicine, Integral Institute of Medical Sciences and Research Centre, Lucknow, IND; 2 Biotechnology, Era University, Lucknow, IND

**Keywords:** drug, type 2 diabetes mellitus, hyperglycaemia, haemoglobin a1c, diabetes mellitus

## Abstract

Purpose: This retrospective observational study aimed to comprehensively analyse the clinical profile and treatment modalities of patients diagnosed with type 2 diabetes mellitus (T2DM) who were treated at a tertiary care centre.

Methods: The study included a cohort of 300 individuals who sought medical care at the hospital from January 2023 to January 2024. The analysis primarily examined parameters such as the mean number of anti-diabetic medications per prescription, the proportion of various categories of anti-diabetic medications prescribed, the predominant class and type of anti-diabetic medications prescribed, and the proportion of anti-diabetic medications prescribed from the essential drug lists.

Results: The age distribution demonstrated that 52.0% of participants were above 60 years old, showcasing a substantial elderly representation. Gender distribution emphasized a male predominance at 65.0%, highlighting potential gender-specific implications in type II diabetes. The blood profile analysis of patients with T2DM revealed a range of values for key parameters. Fasting blood glucose levels ranged from a minimum of 101 mg/dL to a maximum of 359 mg/dL, with a mean of 180.01 mg/dl. The comprehensive analysis of anti-diabetic drug utilization, based on the total number of units prescribed, sheds light on the diverse treatment approaches employed for managing diabetes mellitus (DM). Insulin, comprising 31.3% of the total units, plays a pivotal role in glycemic control, with both regular and biphasic formulations contributing significantly at 26.3% and 9.3%, respectively. Among the 300 patients, the overall utilization of anti-diabetic drugs reveals that 38.7% of individuals are using a combination of insulin with oral anti-diabetic drugs, while 61.3% are relying on oral anti-diabetic drugs alone. The most frequently prescribed drug combinations for diabetes management include sulphonylurea with biguanides, emerging as the most prevalent combination with 22 occurrences.

Conclusion: The study's findings contribute valuable insights into the socio-demographic profiles and anti-diabetic drug utilization patterns among diabetes patients.

## Introduction

Diabetes mellitus (DM) is a widespread metabolic ailment that affects people worldwide, and India bears a substantial burden of this condition. Hyperglycemia occurs due to abnormalities in the metabolism of carbohydrates, fats, and proteins, resulting in errors in the production or action of insulin, or both [1]. The World Health Organization predicts a significant increase in the global incidence of diabetes, with an estimated 300 million cases expected by 2021, compared to 5 million cases in 1995. It is expected that by 2030, India would exceed 100 million diabetes patients. Urban regions are expected to have a prevalence rate of 20%, while rural areas are expected to have a prevalence rate of roughly 10% [2].

Diabetes may be classified into two categories: type 1, which requires insulin for management; and type 2, which does not need insulin for management. Individuals with type 1 diabetes need exogenous insulin for proper management, whereas type 2 diabetes is first addressed with weight loss, adherence to a diabetic diet, and regular exercise. If dietary and lifestyle interventions prove insufficient, pharmacological treatments are considered, including oral medications. In cases where these are inadequate, insulin therapy may be initiated as an alternative if deemed necessary. This comprehensive approach reflects the nuanced management of DM, which often requires tailored strategies to achieve optimal control and health outcomes [3].

Individuals with diabetes in India have unique clinical features in comparison to white people. Significantly, there is an earlier age at which symptoms begin, a lower BMI, and a greater amount of abdominal fat, all of which contribute to insulin resistance. The growing burden of diabetes may be attributed to several reasons, including lack of knowledge, cultural influences, socioeconomic issues, and sedentary lifestyles [4]. Drug consumption studies are essential for comprehending the social consequences of drugs. These studies serve as potent instruments for exploration, laying the groundwork for decision-making in healthcare. The World Health Organization provides drug use indicators that may be included in these investigations. There are several recommendations available for various categories of drugs used in the treatment of diabetes [5,6].

This retrospective investigation examines the clinical characteristics and treatment of type 2 diabetes mellitus (T2DM) at a tertiary care facility. The study is driven by the increasing worldwide incidence of T2DM and the need to get a full understanding of the illness in the intricate tertiary care setting. The research seeks to gain significant insights into the epidemiological landscape of T2DM and the unique nature of the disorder by examining its many clinical features, treatment patterns, and healthcare resource consumption. In addition, studying treatment adherence, complication rates, and temporal patterns may help develop customized treatments, enhance patient outcomes, and provide evidence-based practices. This has implications for both clinical care and healthcare policy in the tertiary care context.

## Materials and methods

This retrospective, observational study was conducted at Integral Institute of Medical Sciences, Department of Internal Medicine, Lucknow, with prior approval from the ethics committee. The study aimed to analyse the prescription pattern of anti-diabetic medications among patients attending the hospital between January 2023 and January 2024. Electronic medical records from the department's system were utilized for data collection. The study population comprised of 300 subjects aged 18 years and above who were receiving anti-diabetic therapy. Informed consent was taken, and patient confidentiality was strictly maintained throughout the study.

Sample size

The sample size was determined based on statistical power calculations to ensure the study could detect significant differences or associations with a high level of confidence. The primary outcome variable was the prescription pattern of anti-diabetic medications. Covariates included patient demographics (age, gender), socioeconomic status, duration of diabetes, comorbid conditions, and previous treatment history. These factors were chosen to comprehensively understand the influences on prescription. 

1) Population Variation: The variability or dispersion of the population regarding the prescription patterns of anti-diabetic medications. 2) Confidence Level: The desired level of confidence (typically 95% or 99%) in the study results. 3) Margin of Error: The acceptable margin of error (usually expressed as a percentage). 4) Expected Effect Size: The expected difference or effect size that the study aims to detect. 5) Statistical Methodology: The statistical method used for analysis (e.g., hypothesis testing, confidence interval estimation).

Given these factors, we used statistical formulas or software tools (like power analysis or sample size calculators) to determine the appropriate sample size. For instance, if the study aims to detect a certain percentage difference in prescription patterns with 95% confidence and a specific margin of error, calculations would be based on these parameters. In the case of this study with a sample size of 300 patients, the determination process likely involved ensuring that this sample size was sufficient to provide statistically meaningful results while considering the factors mentioned above. The goal is to balance statistical power (the ability to detect an effect if it exists) with practical considerations such as feasibility and resources available for data collection and analysis.

Data collection and analysis

Trained personnel extracted relevant information from electronic medical records, including sociodemographic details, details of anti-diabetic drug therapy, and physiological parameters such as BMI, weight, and height. The following parameters were recorded for each participant:

1) Sociodemographic information: This included age, gender, occupation, and residential location of the participants. 2) Anti-diabetic drug therapy: Details of the anti-diabetic medications prescribed were recorded, including the name of the drug, dosage regimen (dose and frequency), duration of therapy, and any co-prescribed medications. The number of anti-diabetic drugs per prescription was noted to calculate the average number of drugs per prescription. 3) Physiological parameters: Anthropometric measurements such as BMI, weight, and height were documented to assess]the baseline health status of the participants.

The collected data were analysed to determine various parameters related to the prescription pattern of anti-diabetic medications:

1) Average number of anti-diabetic drugs per prescription: The total number of anti-diabetic drugs prescribed during the study period was divided by the total number of prescriptions to calculate the average number of drugs per prescription. 2) Percentage of different classes of anti-diabetic drugs prescribed: The total number of prescriptions for each class of anti-diabetic drugs (e.g., sulphonylureas, biguanides, insulin) was calculated, and the percentage of prescriptions belonging to each class was determined. 3) Most common class and type of anti-diabetic drugs prescribed: The class and type of anti-diabetic drugs that were most frequently prescribed were identified based on the frequency of prescriptions. 4) Percentage of anti-diabetic drugs prescribed from essential drug lists: The percentage of anti-diabetic drugs prescribed from the World Health Organization (WHO) Essential Medicines List and the Indian National Essential Drug List was calculated to assess adherence to essential drug lists.

Data analysis for this study was conducted using the Statistical Package for the Social Sciences (SPSS) version 26, developed by SPSS Inc. in Chicago. The significance level was set at p < 0.05, indicating the threshold for determining statistical significance. Categorical data analysis was performed using the Chi-square test, a widely utilized statistical method for assessing associations between categorical variables. This test helped in exploring relationships between different categorical variables, such as the prescription of specific drug classes and patient demographics. The results of the analysis were presented in terms of actual numbers, means, and percentages. Actual numbers provided a clear depiction of the frequency of occurrences, while means offered insights into the central tendency of continuous variables, such as age and physiological parameters. Percentages were utilized to represent proportions relative to the total sample size, facilitating comparisons and interpretations of relative frequencies. By employing SPSS software and utilizing appropriate statistical tests such as the Chi-square test, the study analysed the prescription pattern of anti-diabetic medications comprehensively. The utilization of actual numbers, means, and percentages in result presentation ensured clarity and facilitated a better understanding of the findings, enhancing the interpretability and utility of the study outcomes.

Ethical considerations

The study protocol was reviewed and approved by the ethics committee of the Integral Institute of Medical Sciences, ensuring compliance with ethical standards. Patient confidentiality was maintained by anonymizing the collected data, and all analyses were performed at the aggregate level to protect patient privacy.

## Results

Socio-demographic profile of T2DM patients

Comprehensive findings are detailed in Table 1.

**Table 1:** Socio-demographic profile of T2DM patients T2DM: Type 2 diabetes mellitus

Variables	Characteristics	Number	%
Age	40 to 60 yrs	120	40.0
	above 60 yrs	156	52.0
	less than 40 yrs	24	8.0
Minimum age of the T2DM patients: 24 yrs; maximum age: 81 yrs; mean SD age: 57.75±13.393 yrs			
Gender	Female	105	35.0
	Male	195	65.0
Living status	Rural	122	40.7
	Urban	178	59.3
Occupational status	Employed	45	15.0
	Self-Employed	92	30.7
	Unemployed	163	54.3
Educational status	Graduation	145	48.3
	Illiterate	26	8.7
	Post-Graduation	25	8.3
	Primary School	33	11.0
	Secondary School	71	23.7
Duration of DM	Less than 1 year	18	6.0
	1 to 5 years	130	43.3
	6 to 10 years	107	35.7
	Above 10 years	45	15.0
Family History of DM	No	168	56.0
	Yes	132	44.0
	Minimum	Maximum	Mean SD
Weight	44.0	92.2	68.299±9.0040
BMI	18.5	37.5	27.488±4.2452
Total	300		

Blood profile analysis of patients with T2DM

The blood profile analysis of patients with T2DM revealed a range of values for key parameters. Fasting blood glucose levels ranged from a minimum of 101 mg/dL to a maximum of 359 mg/dL, with a mean of 180.01 mg/dl. Postprandial (PP) blood glucose exhibited a range between 149 mg/dL and 493 mg/dL, with a mean ± SD of 251.39 ±75.93 mg/dL. The HbA1c levels ranged from 6.8% to 13.2%, with a mean ± SD of 9.40 ± 1.8%. These results provide a comprehensive overview of the blood profile of T2DM patients, indicating variations in glucose levels and HbA1c, which are crucial indicators for assessing diabetes management and control (Table 2).

**Table 2:** Blood profile analysis of patients with T2DM PP: Postprandial; T2DM: Type 2 diabetes mellitus

Variables	Minimum	Maximum	Mean	SD
Fasting	101	359	180.01	51.137
PP	149	493	251.39	75.933
HbA1c	6.8	13.2	9.460	1.8438

Overall utilization of anti-diabetic drugs on the basis of total number of units prescribed

The comprehensive analysis of anti-diabetic drug utilization, based on the total number of units prescribed, sheds light on the diverse treatment approaches employed for managing DM. Insulin, comprising 31.3% of the total units, plays a pivotal role in glycemic control, with both regular and biphasic formulations contributing significantly at 26.3% and 9.3%, respectively. The utilization of long-acting insulin at 13.3% underscores its importance in providing sustained blood sugar management. Additionally, oral anti-diabetic agents such as sulphonylurea (48%), meglitinide (28.7%), biguanides (52%), thiazolinidinedione (28.3%), dipeptidyl peptidase 4 (DPP4) inhibitors (49.7%), alpha glucose inhibitors (40%), and sodium-glucose co-transporter (SGLT-2) inhibitors (28.3%) collectively contribute to the multifaceted approach to diabetes treatment. 

Overall utilization of anti-diabetic drugs

**Figure 1:**
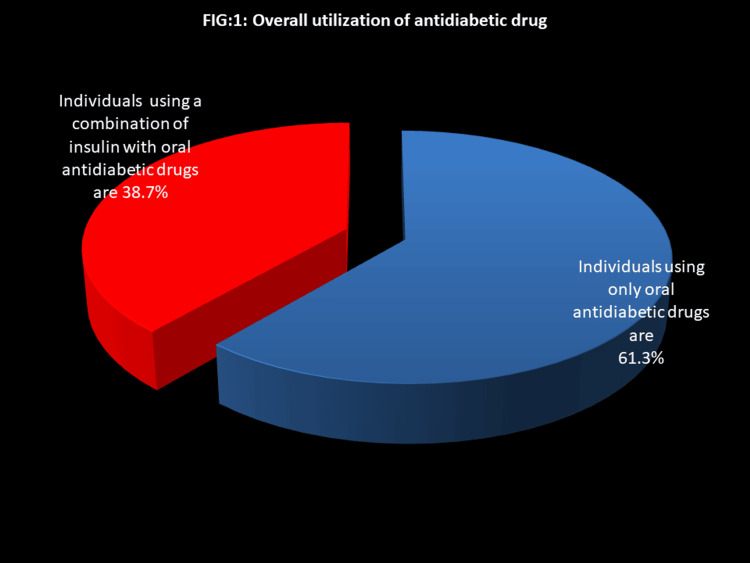
Overall utilization of anti-diabetic drugs Red: Individuals with the combination of insulin and oral anti-diabetic drugs; Blue: Individuals using only oral anti-diabetic drugs

Among the 300 patients, the overall utilization of anti-diabetic drugs reveals that 38.7% of individuals are using a combination of insulin with oral anti-diabetic drugs, while 61.3% are relying on oral anti-diabetic drugs alone (Figure 1).

Prescribing frequency of different class of oral hypoglycaemic agents alone and with insulin

**Figure 2:**
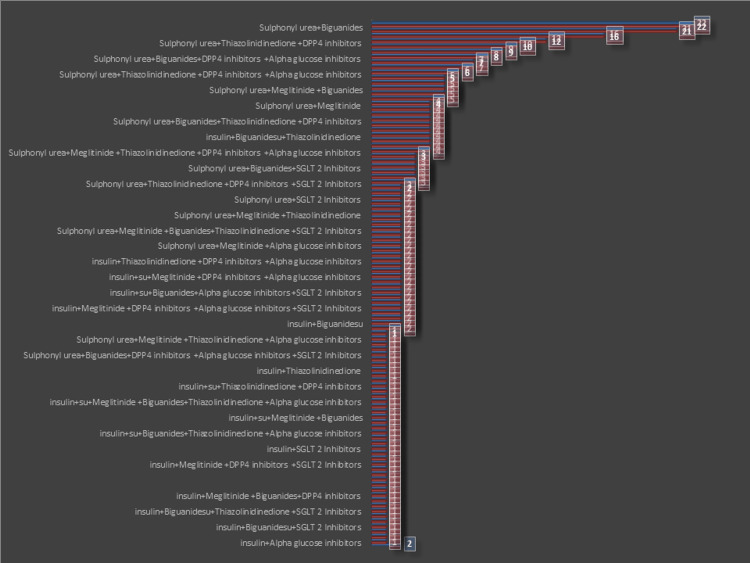
Prescribing frequency of different class of oral hypoglycemic agents alone and with insulin

Our study revealed the two most prevalent combinations of anti-diabetic drugs, with glimepiride and biguanides, and sulphonylureas with biguanides and alpha-glucosidase inhibitors each constituting 22% of cases. Additionally, sulphonylurea with biguanides and DPP4 inhibitors, as well as sulphonylurea with DPP4 inhibitors alone, are highly frequent, with 16 and 12 occurrences, respectively (Figure 2).

## Discussion

In this study involving 300 T2DM patients, an in-depth analysis of socio-demographic profiles revealed notable characteristics. The age distribution demonstrated that 52.0% of participants were above 60 years old, showcasing a substantial elderly representation. Gender distribution emphasized a male predominance at 65.0%, highlighting potential gender-specific implications in T2DM. The urban predominance in living status (59.3%) suggested urban environments as significant factors in diabetes prevalence. A majority of participants (54.3%) were unemployed, underscoring potential socio-economic influences. Educational diversity was evident, with 48.3% having a graduation level. The duration of diabetes varied, with 43.3% experiencing it for one to five years. Weight examination highlighted a mean weight of 68.299 ± 9.0040 kg, emphasizing the need for weight management in diabetes care. These findings provide valuable insights into the intricate landscape of anti-diabetic drug utilization, facilitating a holistic understanding of the varied therapeutic interventions adopted in the management of diabetes. 

The study shows a significant representation of the elderly, with more than half of the participants being over the age of 60. This is consistent with the worldwide trend of an increased incidence of diabetes in older age groups. Furthermore, a significant male preponderance (65.0%) was identified, highlighting possible gender-specific consequences in diabetes. Interestingly, other research, such as those by Abdi et al. and Upadhyay et al., also found a relative male predominance, while investigations in Nepal and Chennai found a relative female preponderance [7-10].

This research offers insight on how urban surroundings and socioeconomic variables influence the incidence of diabetes among individuals in India. The significant urban predominance in living status (59.3%) emphasizes the potential role of urbanization in influencing diabetes rates, which is consistent with the well-established understanding of how urban lifestyles, characterized by specific dietary habits and sedentary behaviors, contribute to an increased prevalence of diabetes. Furthermore, the majority of unemployed participants (54.3%) highlight the considerable socioeconomic impacts on diabetes, which is consistent with recognized connections in the study. The comparison analysis, based on the data of Mohan et al., showed a two to three times greater incidence of diabetes among urban people in India than rural adults prior to 2005 [11]. This is further corroborated by a 2.48 odds ratio from large population surveys done between 2003 and 2005. Ranasinghe et al. performed a thorough investigation that included data from 1,778,706 persons across 69 studies completed from 1972 to 2017, and found strong evidence of a significant increase in diabetes prevalence in both rural and urban India, which was detected independently in both genders [12]. These results highlight the critical need for focused public health interventions that address urbanization-related lifestyle variables and socioeconomic inequalities in order to effectively manage the rising diabetes burden in the Indian population. Diabetes affects people from all educational levels, as seen by the observed educational variety (48.3% have a graduation level). Education may have an impact on health-seeking behavior, adherence to treatment, and lifestyle changes. This emphasizes the significance of tailoring health education campaigns to different groups [13].

The variable length of diabetes, with 43.3% having it for one to five years, demonstrates the dynamic nature of diabetes therapy. Early diagnosis and management are critical for improved results. The mean weight of 68.299 ± 9.0040 kg highlights the importance of weight control in diabetes therapy, since obesity plays a role in disease development [13,14]. The entire blood profile examination showed differences in fasting and PP blood glucose levels, as well as HbA1c values. These differences give a glimpse of the subjects' glycemic control levels. The large range in HbA1c readings (6.8%-13.2%) reflects the severity of diabetes in the study cohort. The study's extensive review of anti-diabetic medicine use demonstrates a varied approach to diabetes management. Insulin is essential for glycemic control, and the wide range of oral anti-diabetic medications used underscores the complexities of diabetes care. The preference for combinations including sulphonylurea and biguanides emphasizes their importance in treatment regimens. When compared to the research by Agarwal et al., the prevalence of sulphonylureas and biguanides remains steady, demonstrating that doctors continue to choose them for controlling T2DM [15]. The study found that a large number of patients (32.5%) had both diabetes and hypertension, offering important insights into diabetics' health profiles. Furthermore, family history appeared as an important predictor, with the vast majority (71.6%) having a favorable family history of diabetes. These results highlight the need of implementing comprehensive management regimens that treat both diabetes and related diseases. Butt et al., Patel et al., and Pitale et al. provide further insights into gender distribution, educational levels, family history, and diabetes duration [10,16,17]. These studies help to broaden the knowledge of the demographic and clinical aspects of diabetes populations in various countries.

Limitations

Several limitations were acknowledged in this study, including its retrospective design, which limited the ability to establish causal relationships. Additionally, the study was conducted at a single center, which may limit the generalizability of the findings to other settings. Furthermore, the accuracy and completeness of electronic medical records relied on the quality of documentation, which could introduce bias or errors in the data analysis.

## Conclusions

Overall, the study's results provide significant knowledge on the socio-demographic characteristics and patterns of anti-diabetic medicine use among individuals with diabetes. The varied attributes found emphasize the need for tailored and comprehensive strategies in the care of diabetes, taking into account individual disparities in age, gender, socio-economic circumstances, and concurrent ailments. The inclusion of comparative research enhances the conversation by offering a wider perspective and emphasizing the differences in diabetes characteristics across other populations and geographical areas. These observations are crucial for customizing efficient diabetic treatment regimens and public health measures.
